# Therapeutic Benefits of Bioactive Compounds in Brain Disorders

**DOI:** 10.2174/011570159X404730250422071304

**Published:** 2025-04-28

**Authors:** Arianna Vignini

**Affiliations:** 1 Department of Clinical Sciences, Faculty of Medicine, Università Politecnica delle Marche, Ancona, Italy

Food Bioactive Compounds (BACs) are naturally occurring substances found in small amounts, particularly in plant-based foods [1]. These compounds are crucial for human health due to their pharmacological and toxicological properties. Increasing scientific evidence shows that many BACs contribute to disease prevention, especially in the case of Non-communicable Diseases (NCDs), including neurological disorders [2]. BACs are diverse in chemical structure and function, which is why they have garnered significant attention for their potential health benefits. As a result, they are widely utilized in the development of nutraceuticals, functional foods, and food additives.

Substantial epidemiological evidence now supports the protective benefits of diets rich in plant-based foods, with the Mediterranean diet (MedDiet) standing out as a key example [3]. The MedDiet, rich in antioxidants, anti-inflammatory, immune-boosting, neuroprotective, antidepressant, antistress, and senolytic properties, plays a critical role in the prevention and management of neurological diseases, as well as in slowing cognitive decline.

This special thematic selection, titled “Therapeutic Benefits of Bioactive Compounds in Brain Disorders”, consists of two individual issues featuring selected reviews and research articles from leading research groups. This issue focuses on dietary components, natural compounds, and medicinal plants that have shown beneficial effects on neurological diseases and brain health.

In the manuscript by Zupo *et al*., the authors conducted a systematic review examining the impact of Oleocanthal (OC) from Extra-Virgin Olive Oil (EVOO) on amyloid-β (Aβ) accumulation in preclinical Alzheimer's disease (AD) models. This study highlights the anti-inflammatory and neuroprotective properties of EVOO biophenols, which are essential elements of the MedDiet. The authors concluded that preclinical data suggest promising effects of OC from MedDiet's EVOO in alleviating the Aβ burden in AD [4]. Another review describes the progress made in researching cholinesterase inhibitors derived from the Buxaceae family, which could serve as promising drug candidates for AD treatment [5]. A key factor contributing to AD is the reduced level of acetylcholine (Ach) in the brain. The cholinergic hypothesis suggests that increasing acetylcholine levels may alleviate AD symptoms, and steroidal and terpenoidal anticholinesterase alkaloids show promise as effective Ach esterase (AChE) inhibitors. Neuroprotectin D1 (NPD1), a mediator derived from docosahexaenoic acid (DHA) and a derivative of α-linolenic acid, also plays a vital role in maintaining neurological health. Classified as a Specialized Pro-resolving Lipid Mediator (SPM), NPD1 has anti-inflammatory and neuroprotective properties essential for the balance of the nervous system. Disruptions in NPD1 signaling are linked to neuroinflammation, oxidative stress, and neuronal cell death, all of which contribute to the progression of neurological disorders. Zia *et al*. [6] thoroughly reviewed NPD1, including its potential therapeutic uses for treating neurological diseases. Similarly, the review by Piccirillo *et al*. examined BACs from plants with sedative and mood-modulating effects, exploring their potential in preventing and/or slowing neurodegenerative processes, such as AD and Parkinson’s disease (PD). Neurodegeneration is driven by multiple biological pathways, and a multifactorial neuroprotective approach targeting various mechanisms holds great promise for disease prevention or just mitigating neurodegeneration [7]. Mood disorders, such as depression and anxiety, are common in individuals with Post-Traumatic Syndrome Disease (PTSD), a psychiatric condition that develops after traumatic events, such as combat, abuse, or accidents. Current PTSD treatments typically involve psychotherapy and medications, but naturally derived compounds present a promising alternative for managing mood disorders with fewer side effects. In their review, Strilbytska *et al*. highlighted the therapeutic potential of several BACs, which have been evaluated in both animal models and human studies for their anxiolytic, antidepressant, and antidementia effects, suggesting their potential for treating PTSD-related mood disorders [8].

PD, a progressive disease marked by both motor and non-motor symptoms, is another area where BACs may offer significant therapeutic benefits. Although the role of the cholinergic nervous system in PD is not yet fully understood, a growing interest in BACs may help illuminate new opportunities for developing therapies targeting the cholinergic system. In another review [9], the authors explored strategies that could involve BACs to alleviate both motor and non-motor symptoms of PD, offering a more comprehensive treatment approach. Lastly, the role of Integrated Stress Response (ISR) signaling in neurological diseases is discussed [10]. Recent research shows that BACs that modulate ISR pathways may become key components of therapies aimed at alleviating the effects of neurological disorders driven by cellular stress. Over the past few years, a growing number of BACs have been identified that modulate ISR pathways, including activators and inhibitors of ISR kinases and agents that affect p-eIF2α dephosphorylation, allowing for a broad strategy of modulation.

In conclusion, the increasing body of research on BACs highlights their significant potential in the prevention and management of neurological diseases. From the neuroprotective effects of compounds in the Mediterranean diet to the therapeutic promise of plant-based bioactive molecules, BACs offer a diverse range of benefits for brain health. As research continues to advance, these compounds may provide novel, effective, and natural alternatives for addressing the growing global burden of neurological disorders.

As the Guest Editor, I would like to express my gratitude to the authors who contributed to this special issue, sharing their research on various aspects of Neuropharmacology related to neurodegenerative diseases. I hope that this special issue serves as a catalyst for further investigation and innovation in this promising field.
